# Development of Natural Compound Molecular Fingerprint (NC-MFP) with the Dictionary of Natural Products (DNP) for natural product-based drug development

**DOI:** 10.1186/s13321-020-0410-3

**Published:** 2020-01-22

**Authors:** Myungwon Seo, Hyun Kil Shin, Yoochan Myung, Sungbo Hwang, Kyoung Tai No

**Affiliations:** 1 Department of Biotechnology, College of Life Science and Biotechnology, Yonsei University, Seoul, Republic of Korea; 2 Bioinformatics and Molecular Design Research Center, Yonsei Engineering Research Park, Seoul, Republic of Korea; 3 Department of Predictive Toxicology, Korea Institute of Toxicology, Daejeon, Republic of Korea; 4 Department of Biochemistry and Molecular Biology, Bio21 Institute, University of Melbourne, Melbourne, VIC 3010 Australia

**Keywords:** Natural product (NP), Natural compound (NC), Dictionary of Natural Product database (DNP), Natural product-based drug development, Molecular descriptor, Virtual screening

## Abstract

Computer-aided research on the relationship between molecular structures of natural compounds (NC) and their biological activities have been carried out extensively because the molecular structures of new drug candidates are usually analogous to or derived from the molecular structures of NC. In order to express the relationship physically realistically using a computer, it is essential to have a molecular descriptor set that can adequately represent the characteristics of the molecular structures belonging to the NC’s chemical space. Although several topological descriptors have been developed to describe the physical, chemical, and biological properties of organic molecules, especially synthetic compounds, and have been widely used for drug discovery researches, these descriptors have limitations in expressing NC-specific molecular structures. To overcome this, we developed a novel molecular fingerprint, called Natural Compound Molecular Fingerprints (NC-MFP), for explaining NC structures related to biological activities and for applying the same for the natural product (NP)-based drug development. NC-MFP was developed to reflect the structural characteristics of NCs and the commonly used NP classification system. NC-MFP is a scaffold-based molecular fingerprint method comprising scaffolds, scaffold-fragment connection points (SFCP), and fragments. The scaffolds of the NC-MFP have a hierarchical structure. In this study, we introduce 16 structural classes of NPs in the Dictionary of Natural Product database (DNP), and the hierarchical scaffolds of each class were calculated using the Bemis and Murko (BM) method. The scaffold library in NC-MFP comprises 676 scaffolds. To compare how well the NC-MFP represents the structural features of NCs compared to the molecular fingerprints that have been widely used for organic molecular representation, two kinds of binary classification tasks were performed. Task I is a binary classification of the NCs in commercially available library DB into a NC or synthetic compound. Task II is classifying whether NCs with inhibitory activity in seven biological target proteins are active or inactive. Two tasks were developed with some molecular fingerprints, including NC-MFP, using the 1-nearest neighbor (1-NN) method. The performance of task I showed that NC-MFP is a practical molecular fingerprint to classify NC structures from the data set compared with other molecular fingerprints. Performance of task II with NC-MFP outperformed compared with other molecular fingerprints, suggesting that the NC-MFP is useful to explain NC structures related to biological activities. In conclusion, NC-MFP is a robust molecular fingerprint in classifying NC structures and explaining the biological activities of NC structures. Therefore, we suggest NC-MFP as a potent molecular descriptor of the virtual screening of NC for natural product-based drug development.
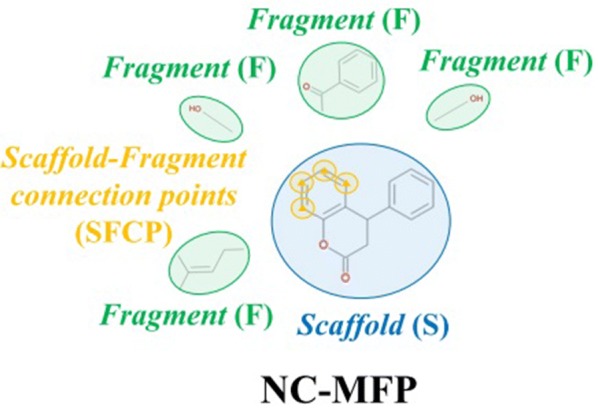

## Introduction

Natural compounds (NC), which are chemical compounds produced by living organisms, have been a significant source of traditional medicine [1]. Usually, plant extracts or herb remedies have been prescribed to treat various afflictions in most countries [2]. Since the known NCs have a wide range of biological activities with structural diversity compared to synthetic compounds, they have been recognized as a valuable resource for pharmaceuticals [3–5].

Since many metabolic pathways are shared among various life forms, thus, life forms may share metabolites with the same or similar molecular structure. Also, NC structures are usually analogous to metabolite [6]. For this reason, NCs are capable of exhibiting various types of physiological activities and thus become an essential source of precursors for new drug development [7]. According to the US Food and Drug Administration (FDA), NCs accounted for 6%, derivatives of NCs accounted for 26%, and mimetics of NCs accounts for 32% of the approved small molecule drugs between 1981 and 2014 [8].

In new drug development, virtual screening is a computational method to find compounds that are likely to exhibit physiological activity in a short time and at low cost using various in silico simulation methods [9]. Since compounds with similar structures may show similar biological activities, an appropriate representation of chemical similarity among compounds is a crucial element for providing high predictability in virtual screening [10, 11]. In chemical structural space described with molecular descriptors as variable axes, the structural similarity among compounds can be expressed as the distance among compounds in the space.

The molecular fingerprint is a way to describe a molecular structure that can convert a molecular structure into a bit string [11, 12]. Since molecular fingerprint encodes the structure of a molecule, it is a useful method to describe the structural similarity among the molecules as a molecular descriptor. Generally, there are two ways of describing a molecular structure with fingerprint; one is substructure key-based fingerprints, and the other is topological path-based fingerprints [13].

The substructure key-based fingerprints represent substructure features of the compound based on the list of structural keys. Molecular ACCess Systems keys fingerprint (MACCS) [14] and PubChem Fingerprints (PubChemFP) [15] are the most commonly used methods substructure key-based fingerprint. MACCS has both 166-bit keyset and 960-bit keyset based on 2D molecular descriptors. These bit keysets were structural keys constructed using SMART patterns and optimized for substructure searching. The 166-bit keyset is the most commonly used and is covered with chemical features related to drug discovery in virtual screening [14]. PubChemFP has generated a binary substructure fingerprint for compound structures. It encoded 881 structural key types that correspond to the substructures for a fragment of all compounds in the PubChem database, which are used by PubChem for similarity neighbor and similarity searching [16].

The topological path-based fingerprints represent all the possible connectivity paths defined by a specific fingerprint via an input compound. AtomPairs2DFingerprint (APFP) [17, 18] is defined in terms of the atomic environment of and shortest path separations between all pairs of atoms in the topological representation of a compound structure [17]. It encodes 780 atom pairs at various topological distances [18]. GraphOnlyFingerprint (GraphFP) [19] is a specialized version of the molecular fingerprint in the chemistry development kit (CDK), which encodes the 1024 path of a fragment in the compound structure and does not take bond order information into account [19].

Most of the molecular fingerprints have been developed to describe molecular structures associated with biological activities based on synthetic compounds. These fingerprints are not usually appropriate for application to NC because the chemical spaces of the biologically active compounds from synthetic and NP do not overlap significantly. Distinctive structural characteristics of NCs as compared to synthetic compounds include a low number of nitrogen atoms, a high number of oxygen atoms, and complex fused ring systems that provide rigid structure and many chiral centers [7, 20–22]. Moreover, since the conventional molecular fingerprints have a small size of fingerprint features, most of the features are included in complex structures like that of the NCs. Hence, it is challenging to represent precise NC structures by conventional molecular fingerprints. Therefore, the novel molecular fingerprint optimized NC structure is necessary to describe the NC structure correctly and to explain the biological activities of the NC structure.

In this paper, we propose a novel molecular fingerprint called “Natural Compound Molecular Fingerprint (NC-MFP).” The NC-MFP represents the structural features of the NCs to explain the biological activity of NC. To fully reflect the structural features and diversity of NCs on the development of the NC-MFP, the NC structures classification system of 16 classes developed by the Dictionary of Natural Product database (DNP) was introduced. The NC-MFP converts structural features of an NC into the bit strings (10,016 bits) with the molecular scaffold, the scaffold-fragment connection points (SFCP), and the molecular fragments of the NC. To comparing the performance of the NC-MFP with other molecular fingerprints, two binary classifications tasks were performed.

## Methods

### Concept of NC-MFP

The structural diversity of compounds synthesized in the course of drug development over the past few decades has been constrained by the structural characteristics of pharmacophores against target proteins and the structure of compounds with biological activities [22]. NCs, on the contrary, may have high structural diversity as they participate in various biological functions, such as agonists or antagonists for enzyme and receptors, signal transduction, protein–protein interaction inhibition, and protein–DNA binding inhibition [23]. In general, since various features of NC structures are related to their biological activities, it is crucial to develop molecular descriptors that can describe the optimal relationship between NC structures and biological activities. Therefore, the first step in developing a molecular fingerprint for a group of NCs involves obtaining information on the structural features of the NCs.

To getting the structural features of NCs, the classification system in the DNP introduced. DNP is a structurally well-classified natural compound database (NCDB) wherein the NCs are categorized into 16 structural classes, according to the representative molecular structures of each group and are classified into sub-groups in each of 16 structural classes [24].

**Fig. 1:**
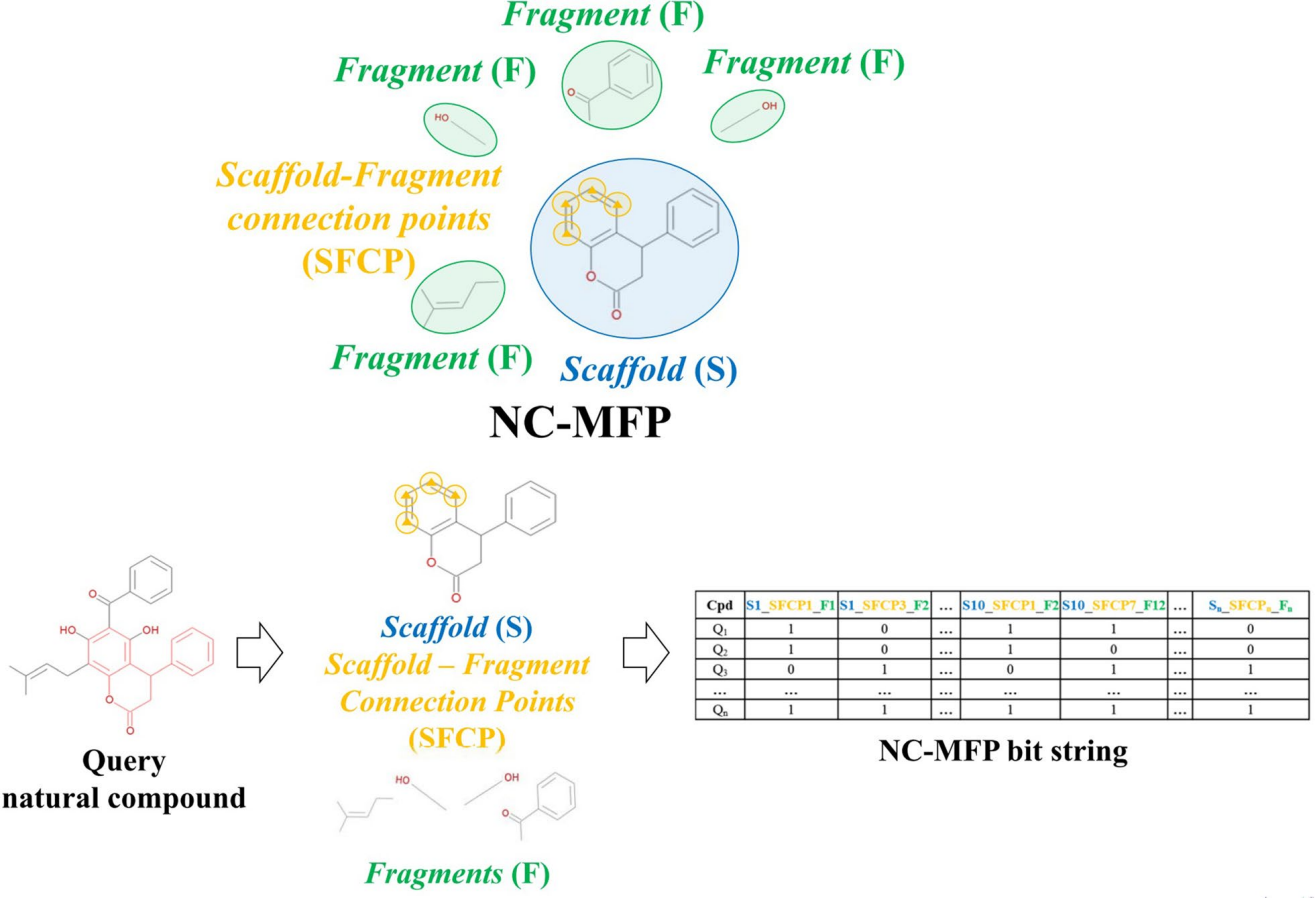
The schematic diagram for the NC-MFP concept is illustrated. The schematic diagram to explain the underlying idea of the hierarchical structure of the NC-MFP is illustrated, a query natural compound is described as a *Scaffold* (blue), *Scaffold-Fragment Connection Points* (yellow), and *Fragments* (green). The NC-MFP of the query natural compound is produced as bit strings with the *Scaffold* (blue), *Scaffold-Fragment Connection Points* (yellow), and *Fragments* (green)

For the representation of structural features of NCs, Scaffolds, Scaffold-Fragment Connection Points (SFCPs), and Fragments were used as the component set that constitutes NC-MFP (Fig. 1). A Scaffold is a part of the chemical structure that is commonly shared between the molecules. Since a specific scaffold can be found among molecules with similar structure or biological activity, Scaffolds provide relevant information to represent NC structures and describe their biological activities [3–5].

SFCPs are the atomic positions on a scaffold where the fragments are connected to the scaffold. Since the changes in the binding position of a functional group in a molecule change its biological activity, SFCPs may play an important role as descriptors in describing the biological activity of NCs.

Fragment refers to a molecular fragment that contains a functional group or groups that are chemically bonded to scaffolds. The biological activity of a molecule varies when a fragment is replaced by another fragment or a combination of fragments on the scaffold.

Since the components, Scaffolds, SFCPs, and Fragments of the NC-MFP are topologically well defined, the NC structures can be represented by bit strings (10,016 bits) (Fig. 1). Since the components of the NC-MFP are the same as those used in Ligand Based Drug Design (LBDD), and SFCPs and Fragments are used to change the biological activity of a reference compound in LBDD. Therefore, the NC-MFP is suitable for describing the relationship between the biological activities and the molecular structures of NCs.

### Molecular scaffolds in NC-MFP

Molecular scaffolds are generally used to describe the common core structures of the molecules [25]. The NCs in DNP are classified into 16 structural classes using the characteristic scaffolds of each group [24]. In medicinal chemistry, a molecular scaffold is used to represent the core structure of a group of active compounds. Since the compounds with the same scaffold may influence a particular metabolic pathway, the molecular scaffolds can effectively contribute to the prediction of biological activities [26].

The scaffold of molecule groups is defined as a common sub-graph of the graphs of the molecule groups. Representatively, Maximum Common Substructure (MCS), Matched Molecular Pairs (MMP), and Bemis and Murko (BM) are the commonly used methods to produce molecular scaffolds [27–31]. The scaffold, as per the MMP method, is defined as the common part among molecules that have different molecular fragments at the same single specific site [28, 29]. MCS method defines a scaffold as the maximum common edge subgraph of the graphs of molecule groups [30]. Unlike the MMP and MCS methods, the scaffolds produced by the BM method reveal a hierarchical structure [31].

**Fig. 2:**
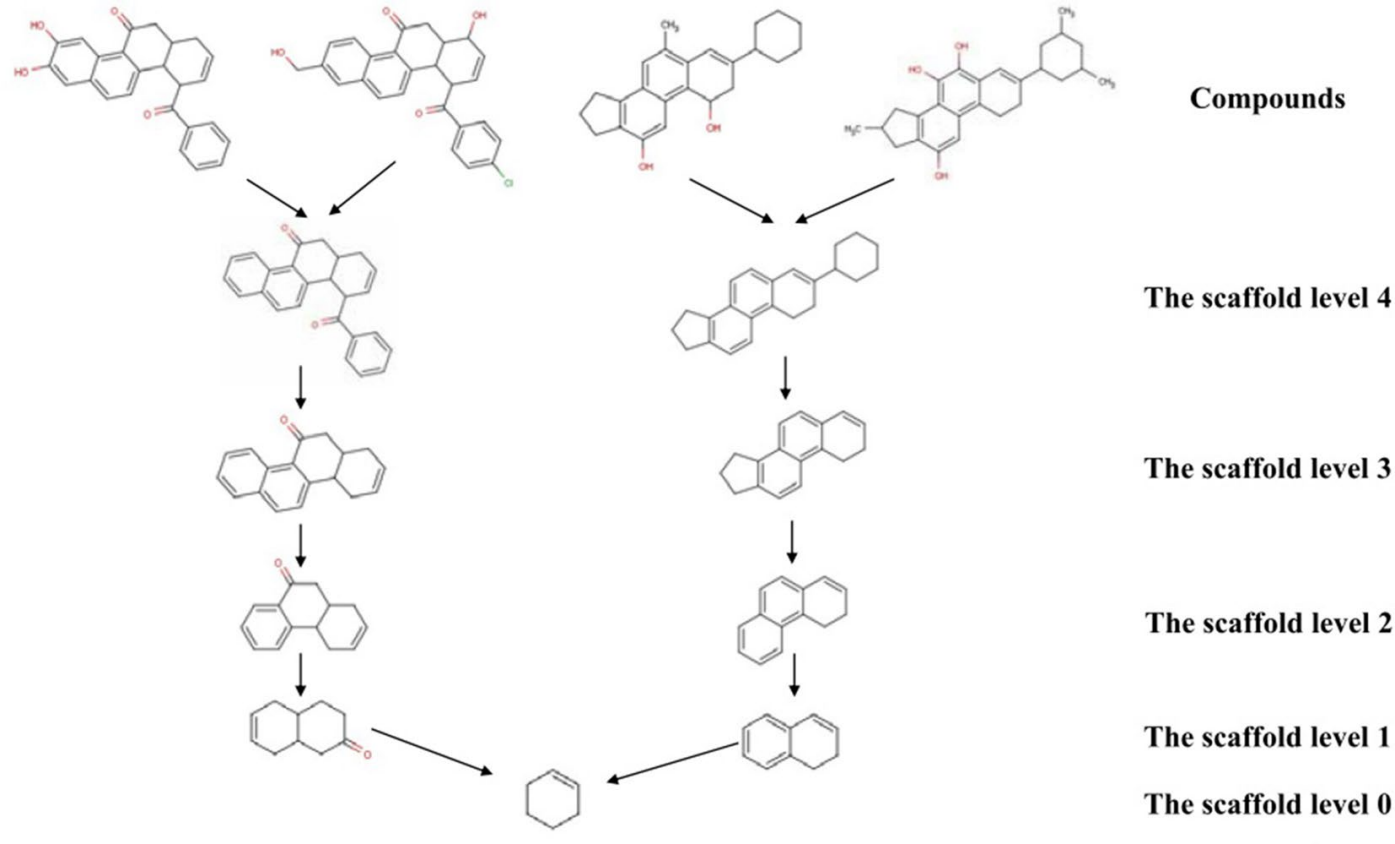
The hierarchical tree of the molecular scaffolds. Based on the Bemis and Murko (BM) scaffold method, functional group of compounds removed. And then the ring systems in the molecular scaffolds are iteratively removed until an only single ring remains. In the hierarchical tree, each node means the molecular scaffolds and assigns a level based on the node position in the tree

Since this study aimed at developing a molecular fingerprint, NC-MFP, that can express the structure of natural products based on the classification system of the DNP, the BM method was used to produce the hierarchical scaffold tree that matched well with the DNP classification system. Using the BM method, a molecular scaffold is produced from a molecular structure by removing the functional groups, while keeping all the rings and the linkers between the rings. The exocyclic double bonds and terminal double bonds are regarded as part of the molecular scaffold. The pruning procedure iteratively generates the molecular scaffolds until only a single ring remains [26]. A level is assigned to each scaffold with its node position at the molecular scaffold-based hierarchical tree. Figure 2 shows the assignment of the level of the scaffolds in the hierarchical tree. The smallest scaffold contains a single ring and is assigned the scaffold level of 0. Since the smallest scaffolds contain a single ring, the NC-MFP can be used only for the compound with at least one ring in a compound. The generation of scaffolds in NC-MFP was implemented in Pipeline Pilot (2017 version) [32].

In DNP, all the NCs are classified into 16 classes, and for each class, a group of structurally representative compounds is presented. NC-MFP constructs a multilayer hierarchical scaffold tree for each DNP class by applying the BM scaffold procedure with the representative molecular structures of each class. Therefore, each class of the DNP consists of a scaffold library with a hierarchical tree structure. Each scaffold in the library is assigned a level.

In other words, the scaffold library (*SL*) of class *i* of the DNP, ${SL}_{i}$ is represented as,

1 $${SL}_{i}\to \left\{\left({s}_{0,1}^{i},{s}_{0,2}^{i},\ldots \right)\right., \left({s}_{1,1}^{i},{s}_{1,2}^{i},\ldots \right), \left({s}_{2,1}^{i},{s}_{2,2}^{i},\ldots \right), \left({s}_{3,1}^{i},{s}_{3,2}^{i},\ldots \right),\ldots \}$$

where ${s}_{j,k}^{i}$ represents the kth scaffold at scaffold level *j* of the DNP class *i*.

### Selection of the optimum NC-MFP scaffold level that gives maximum discrimination

**Fig. 3:**
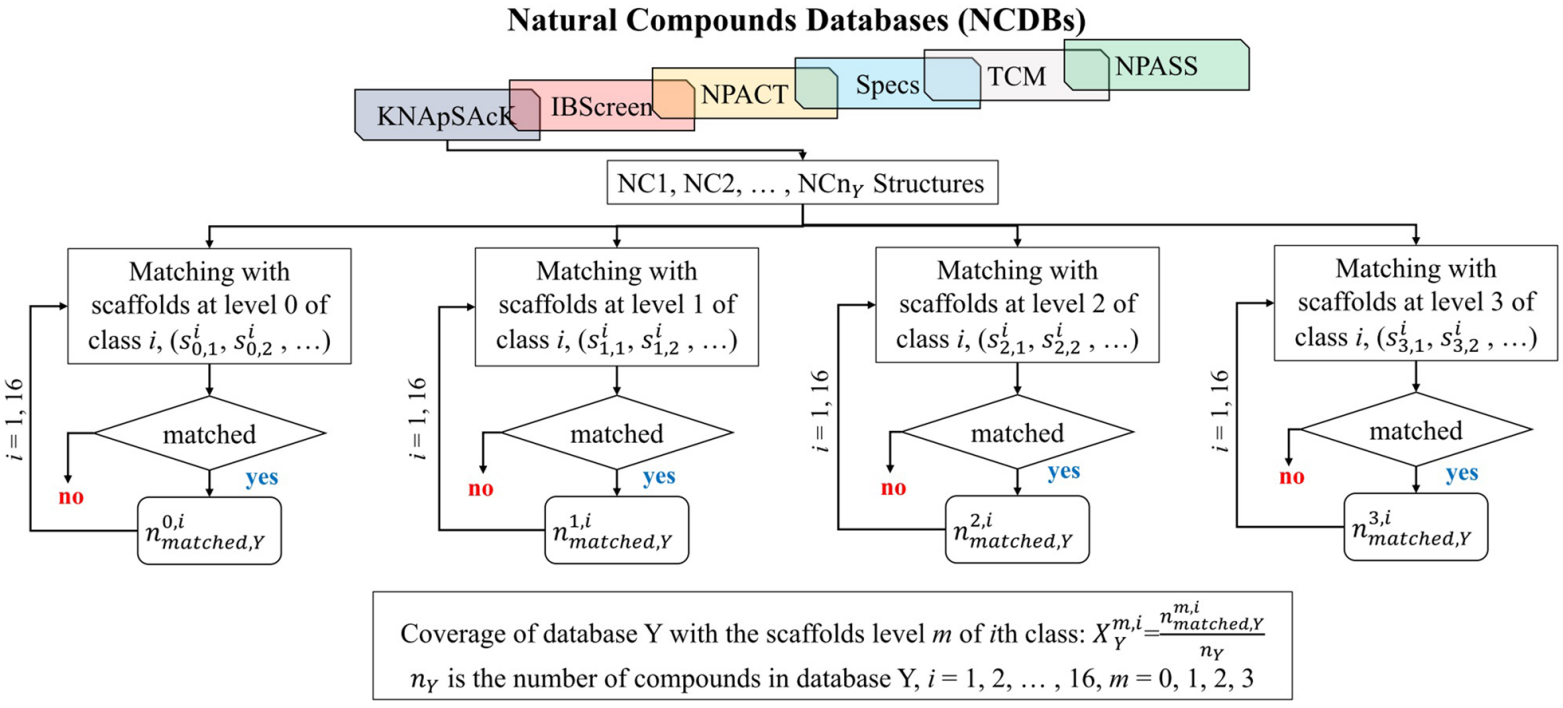
The DB coverage calculation. The DB coverage of molecular scaffolds was calculated according to scaffold levels of from 0 to 3 by using the NCDBs

**Fig. 4:**
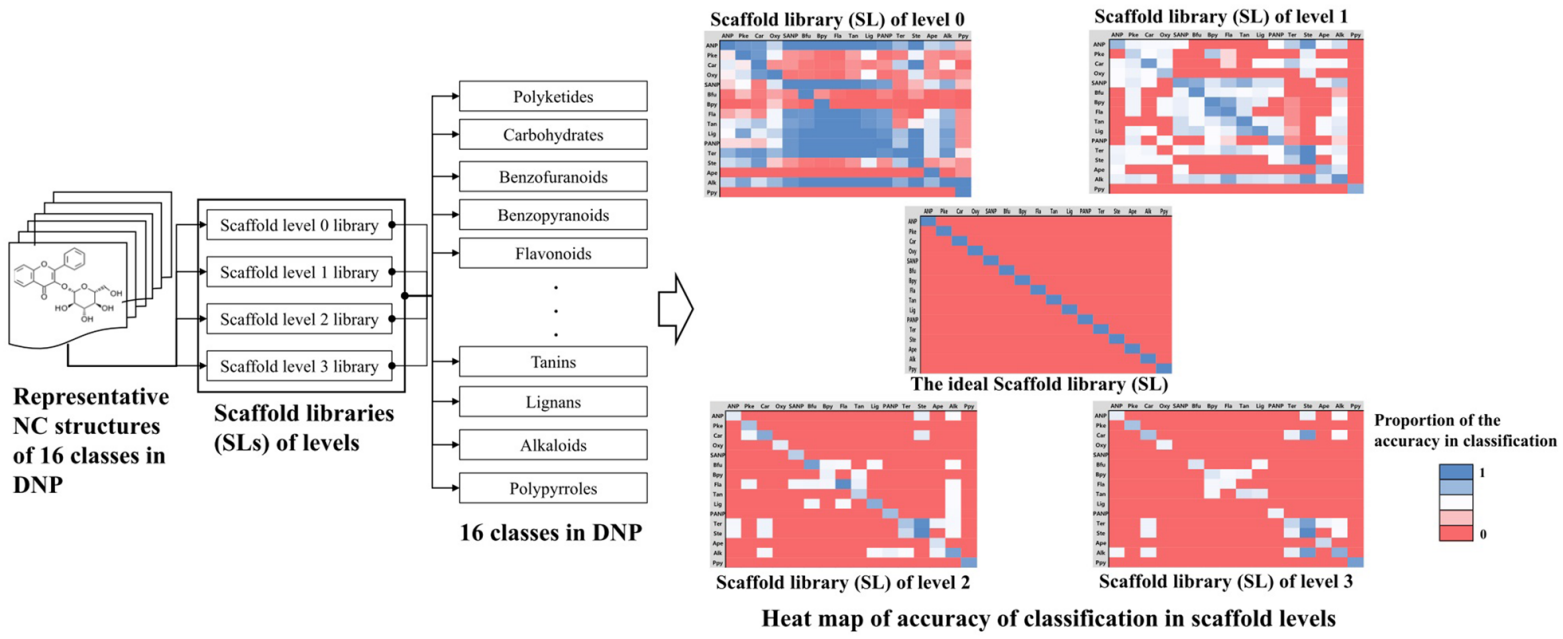
The heat map of the accuracy of classification according to the scaffold levels. The heat map shows that the assignment to NC structures of DNP into 16 classes in DNP by using scaffold library of levels of from 0 to 3. The value is the proportion of the accuracy in classification and ranges from 0 to 1. The best value is closing to 1. The abbreviation of 16 classes is in Table 2

The molecular scaffolds were generated using the molecular structures of the representative compounds from each group in the DNP and were assigned a scaffold level from 0 to 3. To select the scaffold levels with maximum discrimination among the compounds belonging to different classes in the DNP, DB coverage and the accuracy of classification of the scaffolds were calculated at each scaffold level from 0 to 3 by using the Pipeline Pilot 2017 [32]. The DB coverage of a certain scaffold level is defined as the fraction of assigning a NC in Natural Compound Databases (NCDBs) to any of the 16 classes of the DNP using the scaffolds of a certain level by structure matching (Fig. 3). The accuracy of classification of a certain scaffold level is defined as the fraction of correct assignment of an NC to 1 of 16 classes in DNP, where the NC originally belongs (Fig. 4).

To construct an integrated NCDB, we collected all the compounds from several NC databases, KNApSAcK [33], InterBioScreen (IBScreen) [34], Naturally occurring Plant-based Anticancerous Compound-Activity-Target Database (NPACT) [35], Specs [36], Traditional Chinese Medicine (TCM) [37], and Natural Product Activity and Species Source Database (NPASS DB) [38]. Then, the collected compounds were filtered by the criteria that a compound contains at least one ring. The final NCDB consists of 41,082 NCs from KNApSAcK, 56,942 NCs from IBScreen, 1,335 NCs from NPACT, 844 NCs from Specs, 33,902 NCs from TCM, and 24,815 NCs from NPASS, with the total number of NCs in the NCDBs calculated to be 158,920 (Additional file 1).

### NC-MFP generation

The procedure for generating the NC-MFP of a natural compound consists of six steps: I. Preprocessing step, II. Scaffold matching step, III. Fragment list generation step, IV. Scaffold-fragment connection point (SFCP) assigning step, V. Fragment identifying step and, VI. Fingerprint representation step.

**Fig. 5:**
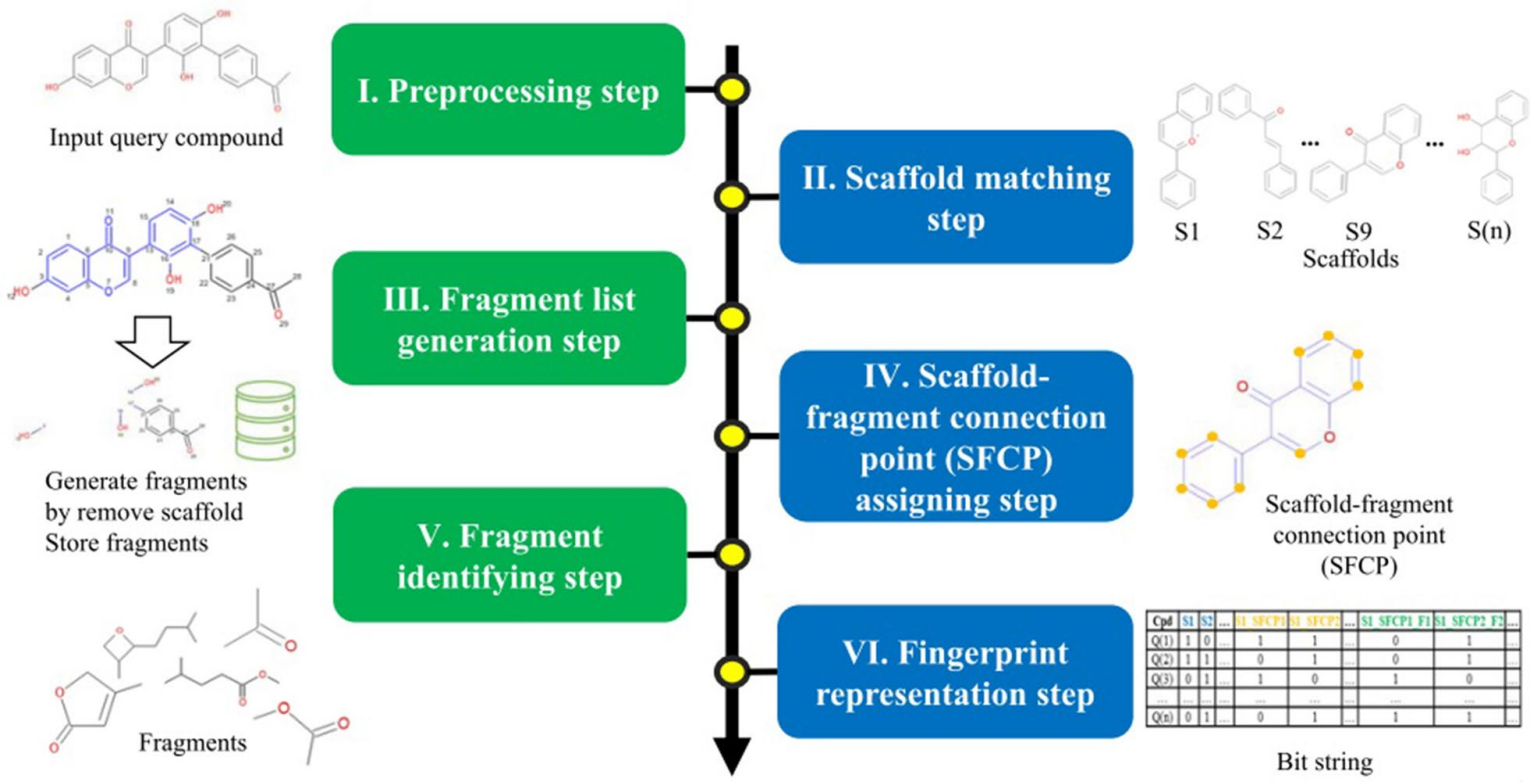
Workflow to generate the NC-MFP. The NC-MFP algorithm consists of six steps. Preprocessing step prepares input query compound for NC-MFP calculation. Scaffold matching step is to find related scaffold from query compounds. Fragment list generation step is to generate fragments by remove scaffold from the input query compound. Scaffold-fragment connection point (SFCP) assigning step is to identify the location on the fragment in the scaffold. Fragment identifying step is to find the fragment information of query compound structure from all fragment list. Fingerprint representation step describes the feature of NC-MFP by a bit string

**Fig. 6:**
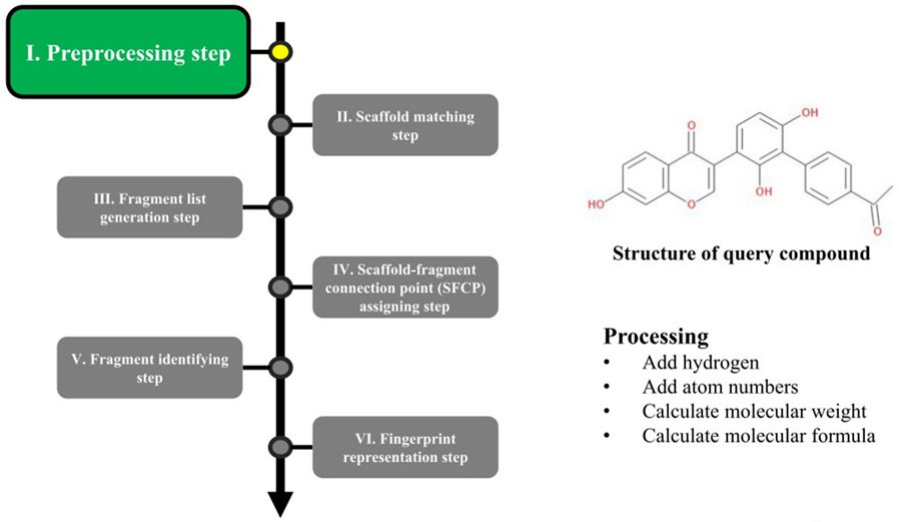
Preprocessing step in NC-MFP algorithm

**Fig. 7:**
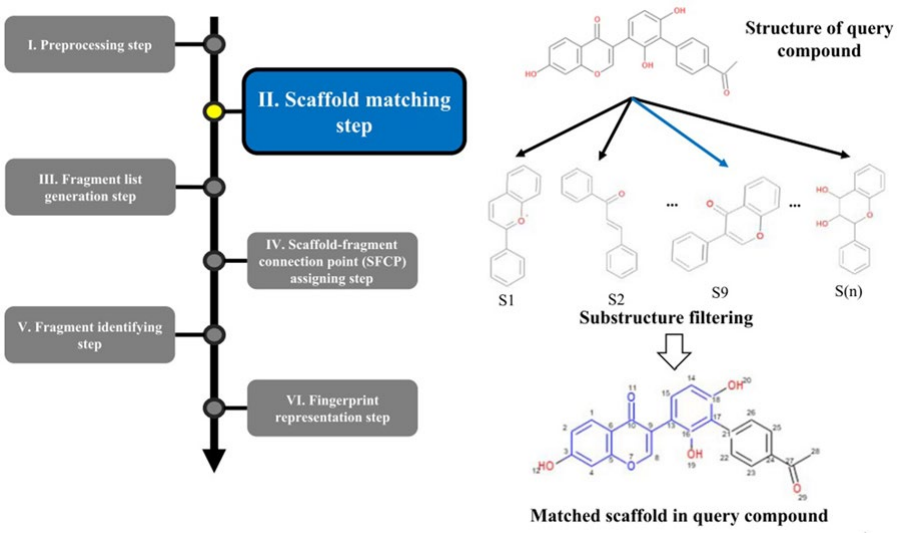
Scaffold matching step in NC-MFP algorithm

**Fig. 8:**
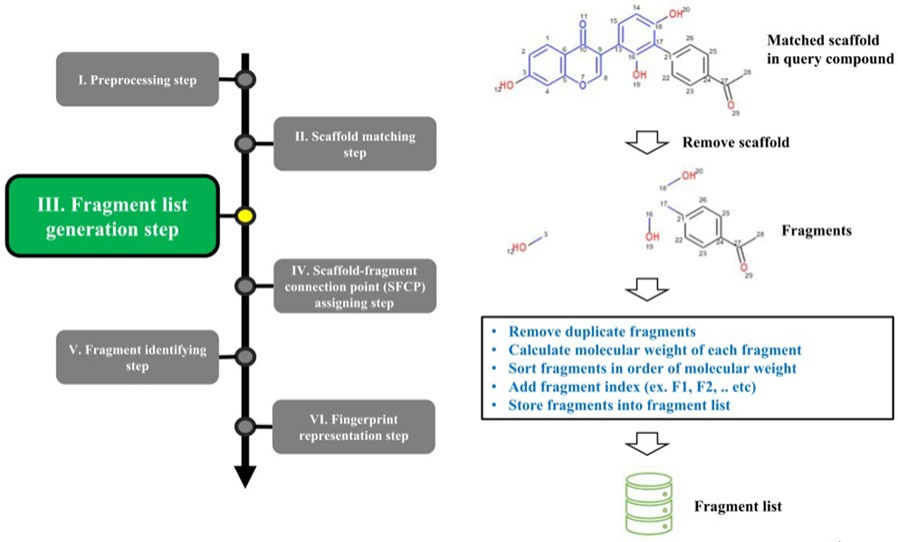
Fragment list generation step in NC-MFP algorithm

**Fig. 9:**
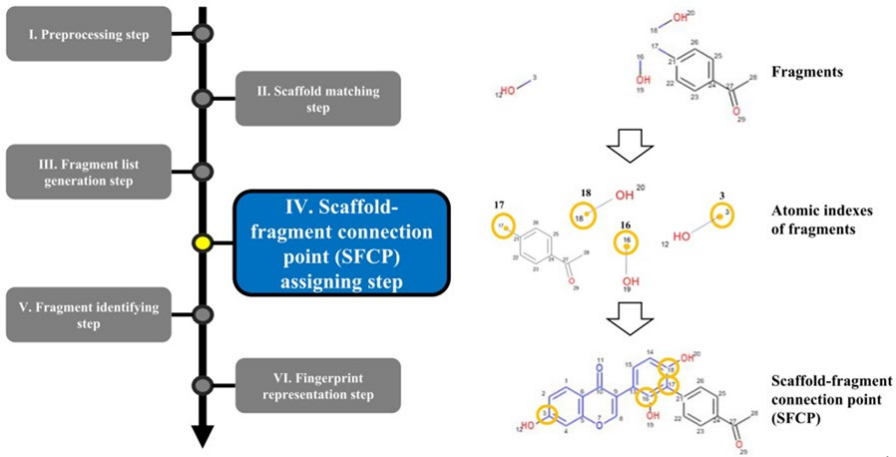
Scaffold-fragment connection point (SFCP) assigning step in NC-MFP algorithm

**Fig. 10:**
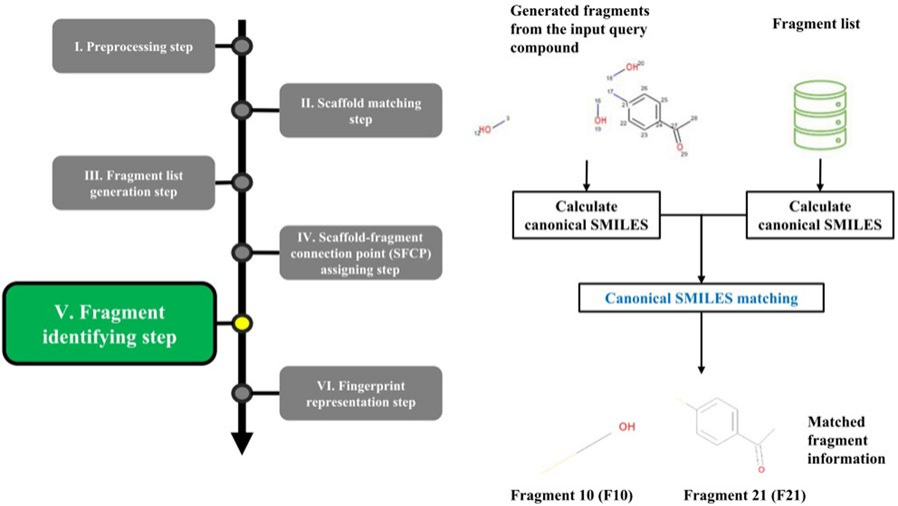
Fragment identifying step in NC-MFP algorithm

**Fig. 11:**
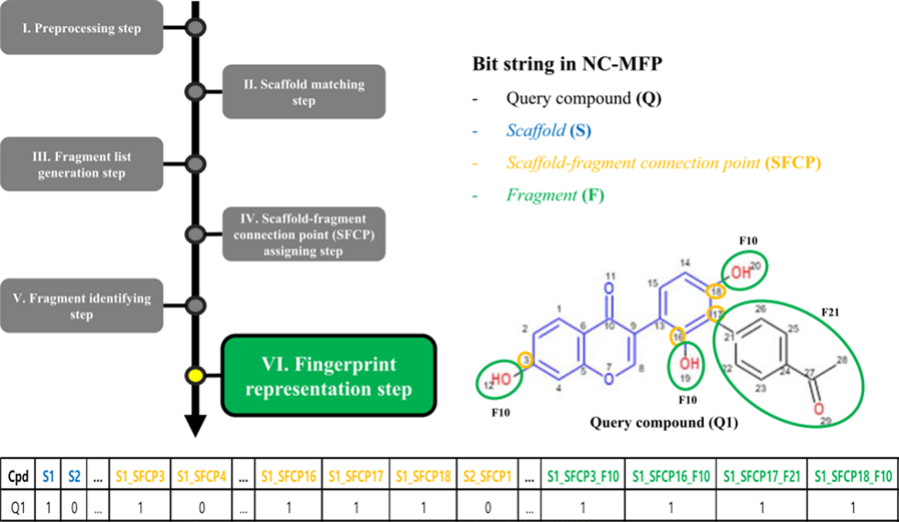
Fingerprint representation step in NC-MFP algorithm

The overall procedure to generate the NC-MFP is described in Fig. 5. At step I, missing hydrogen atoms are added to a query compound, and then, atomic indices are assigned to all the atoms of the compound. Also, molecular properties, such as molecular weight and molecular formula are calculated (Fig. 6). In step II, a scaffold from the scaffold libraries is selected using the substructure filter that uses an exact matching between scaffold and query compound structure (Fig. 7). Step III involves the generation of all fragments by removing the matched scaffold from the query compound. Among all the fragments, duplicated fragments are removed. And then, the molecular weight of each fragment is calculated, which is stored to the fragment list by adding a fragment index in order of molecular weight (Fig. 8). In step IV, the scaffold-fragment connection point (SFCP) on the scaffold is identified as the atomic index assigned to each fragment from the query compound (Fig. 9). In step V, fragments generated from the input query compound are identified by comparing the same with the fragment list. In this process, fragments are converted to canonical SMILES for identification with a fragment of input query compound from the fragment list (Fig. 10). Lastly, in step VI, the fingerprint is represented by the bit string, which is generated based on the scaffold, SFCP, and fragment (Fig. 11).

The NC-MFP algorithm was implemented in Pipeline Pilot (2017 version) [32] (Additional file 2).

### Introduction of popular molecular fingerprints for comparison

To objectively judge the performance of the developed NC-MFP method, widely used molecular fingerprints were introduced to compare classification performance among various methods.

Molecular ACCess System keys Fingerprint (MACCS) [14], PubChem Fingerprint (PubChemFP) [16], AtomPairs2D Fingerprint (APFP) [17, 18], and Graph Only Fingerprint (GraphFP) [19] have been widely used for developing in silico biological activity prediction models by Quantitative Structure–Activity Relationship (QSAR) method as the molecular descriptor. They have been broadly applied to synthetic organic compounds and show excellent performance.

The PaDEL-descriptor program was used to calculate molecular fingerprints [39].

### k-Nearest Neighbors algorithm

Since the k-Nearest Neighbors algorithm (k-NN) is the instance-based algorithm, wherein the performance of binary classification is solely dependent on the molecular descriptor [40], it was used for binary classification to test molecular fingerprint discrimination potential. The k-NN algorithm assigns a data point to a particular class according to the class of k number of nearest neighbor(s) [41, 42]. To search the nearest neighbors, the Tanimoto coefficient [43] was measured with a bit string of the molecular fingerprint. The number of nearest neighbor (k) was assigned the value of 1. Since 1-NN has been used as a reference method to evaluate molecular fingerprints in the previous study [42], the performance of classification tasks using 1-NN was used to compare NC-MFP and other molecular fingerprints. Furthermore, since the 1-NN method only assigned to the class of a single nearest neighbor, it is possible to test the maximized capability of molecular fingerprint as a molecular descriptor in a binary classification task. RapidMiner Studio 9.2 was used to calculate a binary classification by using the k-NN model [44].

### Y-randomization

The Y-randomization test was performed to validate the uniqueness of the model in tasks [45]. The process of the Y-randomization test is as follows. First, endpoint values were randomly shuffled, and then model training processes were repeated on the reshuffled data. Matthews Correlation Coefficient (MCC) [46] and Accuracy (ACC) [47] were calculated from random models developed in each round of Y-randomization. Z-scores were calculated as,

2 $${Z}_{MCC}=\frac{{MCC}_{ori}- {MCC}_{rand}^{mean}}{{\sigma }_{rand}^{MCC}}$$

3 $${Z}_{ACC}=\frac{{ACC}_{ori}- {ACC}_{rand}^{mean}}{{\sigma }_{rand}^{ACC}}$$

${MCC}_{ori}$ or ${ACC}_{ori}$ are MCC or ACC of the original model that were trained with correct biological activity (IC50) values, respectively. ${MCC}_{rand}^{mean}$ or ${ACC}_{rand}^{mean}$ and ${\sigma }_{rand}^{MCC}$ and ${\sigma }_{rand}^{ACC}$ are mean and standard deviation of MCC or ACC values from random models, respectively. If the Z-score of the model is higher than 3, then the model with original data is unique and statistically significant against those developed with random data.

### Binary classification tasks for comparing the performance among some popular molecular fingerprints

**Fig. 12:**
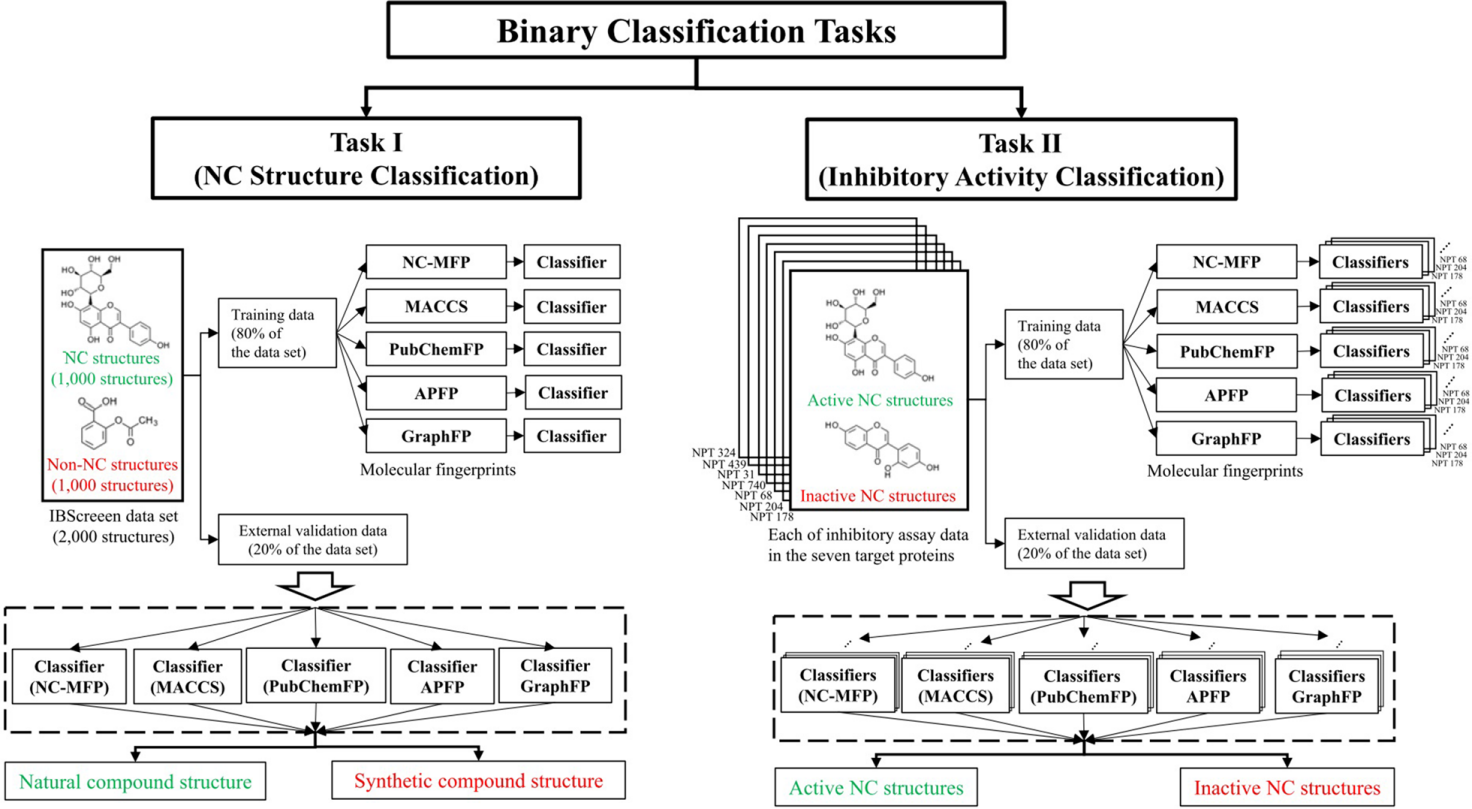
Two types of binary classification tasks

In order to compare the discriminating performance of the NC-MFP with those of some popular molecular fingerprint methods, such as MACCS, PubChemFP, APFP, and GraphFP, two kinds of binary classification tasks were performed; task (I) classification of compounds in commercial library DB into NC or synthetic compound, and task (II) classification of whether a compound is biologically active or inactive for a specific target protein (Fig. 12). The 1-Nearest Neighbors algorithm (1-NN) was used for the binary classifications.

For task I, a data set containing both NCs and synthetic compounds, with 1000 compounds for each class, was constructed from InterBioScreen (IBScreen) [34] database by random selection (Additional file 3). In task I, the accuracy of molecular fingerprints in the classification corresponded to the correctly classified fraction into the NC group or synthetic compound group (Additional file 4). Some classification research of NC structures in the compounds has been performed, such as task I [48].

For task II, seven target proteins and the biologically active and inactive NCs for each target protein were collected from Natural Product Activity and Species Source Database (NPASS DB) [38] as a data set. (Table 1) If experimentally obtained biological activity (IC50) of a compound was less than 10 µmol, then the compound was considered active compound; otherwise, it was deemed inactive compound. The selection of the target proteins from NPASS DB required that the target protein had data for more than 75 experimental inhibitory activities. The criteria for the number of compounds (75) was determined by a trade-off between the number of available target proteins in NPASS and the minimum number of inhibitory activity data required for training and validation of the binary classification task. Seven target proteins satisfied the criteria of more than 75 experimental validations, and 897 NCs corresponded to the seven target proteins (Additional file 5). In task II, the performance among the molecular fingerprints was compared as the accuracy of correctly classify a NC into an active NC or inactive NC (Additional file 6).

**Table 1:** The number of active and inactive compounds for each target protein are summarized

Biological activity	NPASS^b^ Target ID	No. of active compounds^c^	No. of inactive compounds^d^	No. of total compounds with ring structures^e^	No. of total compounds without ring structures^f^
Protein-tyrosine phosphatase 1B inhibitors	NPT178	81	171	252	3
Acetylcholinesterase inhibitors	NPT204	54	108	162	4
Aldose reductase inhibitors	NPT68	57	68	125	3
Beta-secretase 1 inhibitors	NPT740	35	73	108	1
Cyclooxygenase-2 inhibitors	NPT31	31	62	93	1
Butyrylcholinesterase inhibitors	NPT439	28	53	81	1
Cyclooxygenase-1 inhibitors	NPT324	27	49	76	0

Seven target proteins were selected from NPASS DB^a^ together with active and inactive compounds for each target protein

^a^From Natural Product Activity & Species Source Database (NPASS DB), seven biological activities along with related protein targets were selected

^b^Target ID code of the NPASS DB with which one can access protein information (“NPASS Target ID”)

^c^The number of active natural compounds with ring structures obtained with the experimental inhibitory assay (“No. of active compounds”)

^d^The number of inactive natural compounds with ring structures obtained with the experimental inhibitory assay (“No. of inactive compounds”)

^e^The total number of natural compounds with ring structures used for the model development (“No. of total compounds with ring structures”)

^f^The total number of natural compounds without ring structures (“No. of total compounds without ring structures”)

In each of the tasks, 80% of the data set was used for training data. The remaining 20% of the data set was used for external validation data. In task I, the training and external validation data were randomly selected ten times from the data set. Each data for task II was randomly selected ten times from the data set in each of the target proteins. (Fig. 12) The training of the two tasks was performed ten times by tenfold cross-validation, and the external validation was performed ten times. The performance of the molecular fingerprints was evaluated to the average of the ten external validation results from the two tasks.

## Results and discussion

### Generation of NC-MFP scaffold library

As described by Eq. 1, the NC-MFP scaffold library consists of libraries with 16 classes, and each class consists of a scaffold library of level 0, level 1, level 2, and level 3, respectively, with the DNP consisting of representative compounds for each class. The scaffold library for level 0, level 1, level 2, and level 3 was generated with the molecular structures of the representative compounds of each class using the BM method described in Fig. 2. In Table 2, the 16 classes of the DNP are listed with the number of the representative compounds (Additional file 7). The number of scaffolds at each level of each class are summarized. The structural diversity of scaffolds increases according to the increase in the number of representative compounds of the class.

**Table 2:** The classes of the Dictionary of Natural Products (DNP) and scaffold levels are listed

No	Class^a^	Class designation	No. of representative compounds^b^	No. of scaffolds (Lv0)^c^	No. of scaffolds (Lv1)^d^	No. of scaffolds (Lv2)^e^	No. of scaffolds (Lv3)^f^
1	Aliphatic natural products	ANP	31	16	10	4	3
2	Alkaloids	Alk	303	107	177	218	190
3	Aminoacids and peptides	Ape	13	9	9	7	5
4	Benzofuranoids	Bfu	11	5	6	6	3
5	Benzopyranoids	Bpy	15	7	8	4	4
6	Carbohydrates	Car	30	10	13	14	10
7	Flavonoids	Fla	19	8	8	10	2
8	Lignans	Lig	20	9	10	9	1
9	Oxygen heterocycles	Oxy	12	8	7	3	1
10	Polycyclic aromatic natural products	PANP	13	6	8	8	3
11	Polyketides	Pke	12	10	9	11	8
12	Polypyrroles	Ppy	6	6	6	6	6
13	Simple aromatic natural products	SANP	18	9	10	7	0
14	Steroids	Ste	17	5	5	5	6
15	Tannins	Tan	21	8	8	9	6
16	Terpenoids	Ter	141	34	33	28	14
Total			682	257	327	349	262

DNP are listed with its’ designated name. The number of representative compounds of each class are listed. The number of scaffolds at level 0, 1, 2, and 3 are summarized

^a^From Dictionary of Natural Product database (DNP), 16 classes were introduced

^b^The number of representative natural compounds in each group of the DNP (“No. of NC representative group in DNP”)

^c^The number of scaffold level 0. (“No. of scaffolds (Lv0)”)

^d^The number of scaffold level 1. (“No. of scaffolds (Lv1)”)

^e^The number of scaffold level 2. (“No. of scaffolds (Lv2)”)

^f^The number of scaffold level 3. (“No. of scaffolds (Lv3)”)

### Optimization of NC-MFP scaffold level of NC-MFP

With the scaffold libraries summarized in Table 2, the DB coverage and the accuracy of classification of the scaffolds were calculated at each scaffold level from 0 to 3. To select the optimum scaffold level of NC-MFP, which has maximum discrimination power for NC structures, we analyzed the DB coverage and the accuracy of classification results. The DB coverage was calculated as per the procedure described in Fig. 3, and the results are summarized in Table 3.

**Table 3:** The result of DB coverage

The DB coverage of the molecular scaffolds [${X}_{Y}^{m}$, (%)]				
NCDBs (Y)	Level 0 (${X}_{Y}^{0}$)	Level 1 (${X}_{Y}^{1}$)	Level 2 (${X}_{Y}^{2}$)	Level 3 (${X}_{Y}^{3}$)
KNApSAcK	99.95	75.70	43.08	12.79
IBScreen	99.96	79.49	22.07	3.43
NPACT	100.00	80.67	54.31	18.13
Specs	99.88	85.78	64.69	33.29
TCM	99.98	74.39	34.99	13.38
NPASS	99.97	72.24	37.52	13.10
Avg. performance	99.96	78.05	42.77	15.69

The natural compound databases (NCDBs) coverage defined by Eqs. (2) and (3) are summarized at different scaffold levels

“NCDBs” means Natural Compound Databases. “Avg. performance” means the average value of performance

The coverage of a database Y using level m scaffolds was calculated as,

4 $${X}_{Y}^{m}=\sum_{i=0}^{16}{X}_{Y}^{m,i}$$

5 $${X}_{Y}^{m,i}=\frac{{n}_{matched, Y}^{m,i}}{{n}_{Y}}$$

where *i* represents the *i*th class of the DNP, ${n}_{Y}$ is the number of compounds in database Y, and ${n}_{matched, Y}^{m,i}$ is the number of matched compounds with level *m* scaffold in *i*th class. If an NC in database Y belongs to more than two classes of the DNP, then the NC is considered being included in one class only and removes from another class. Table 3 summarizes *DB coverage* with level *m*, ${X}_{Y}^{m}$. The average ${X}_{Y}^{0}$ is 99.96% and the average of ${X}_{Y}^{1}$, ${X}_{Y}^{2}$, and ${X}_{Y}^{3}$ were 78.07%, 42.09%, and 15.73%, respectively. As the level of scaffold set increases, the coverage decreases rapidly. In order to apply NC-MFP to any of NCs with at least one ring in a molecule, the ideal coverage should close to 100% at the scaffold set in NC-MFP. If the molecular structure of all known NCs is used to produce the scaffold set at each level and for each class, then the coverage would be close to 100%, but in this case, a large number of scaffolds would be selected and unsuitable for characterizing each natural product class. However, the coverage would be increased if more representative compounds were introduced to each class of DNP in addition to the representative compounds listed in DNP. It seems that the representative compounds listed for each class from the description of NP structures in DNP are were not enough for covering NCs discovered to date.

Figure 4 describes the classification procedure to check whether the representative compounds in DNP correctly classify to the class which the compound originally belongs to and calculate the *accuracy of the classification* of the scaffold levels. The *accuracy of classification* was obtained with the scaffolds at different scaffold levels of level 0, level 1, level 2, and level 3. The results are shown as heat maps in Fig. 4. The heat map matrix is asymmetric and the element and proportion, of the heat map at level m, ${P}_{i,j}^{m}$, is calculated as

6 $${\text{For diagonal element}} \to {P}_{i,i}^{m}=\frac{{n}_{i}^{m}}{{n}_{i}^{o}}$$

7 $${\text{For off diagonal element}} \to {P}_{i,j}^{m}=\frac{{n}_{i\to j}^{m}}{{n}_{i}^{o}}$$

where ${n}_{i}^{o}$ and ${n}_{i}^{m}$ are the number of the compounds in class *i* and the number of compounds which are correctly assigned to class *i* in level *m*, respectively. ${n}_{i\to j}^{m}$ is the number of compounds assigned to class *j* which originally belonged to class *i*. The ideal heat map, therefore, has all the ${P}_{i,i}^{m}$ are 1 (blue) and all the ${P}_{i,j}^{m}$ are 0 (red). Level 0 and level 1 scaffold library showed poor classification ability, whereas level 2 and level 3 scaffold library showed much better classification than level 0 and level 1 scaffold library; however, the classification was not satisfactory.

The first objective of this study was to determine the optimal scaffold level for the NC-MFP by comprehensively analyzing the results of the *DB coverage* and *accuracy of classification*. The second objective was to find a way to increase the coverage and the accuracy of classification for the NCs in NCDBs based on this analysis.

Since we introduced the DNP’s NC classification system with 16 classes, and the representative compounds of each class for developing NC-MFP using the BM method, the DB coverage of NCs and the accuracy of classification depended entirely on the contents of the DNP. Currently, DNP content and the optimum scaffold level was assigned 2, as a result of careful consideration with both the DB coverage and accuracy in the classification listed in Table 3 and Fig. 4. Scaffold level 2 and level 3 showed similar accuracy in classification, whereas scaffold level 2 showed much bigger DB coverage (Table 3), than that of scaffold level 3. The coverage of scaffold level 2 was too low for practical applications. This disadvantage can be overcome to some extent by using level 1 scaffolds to complement level 2 scaffolds. Hence, scaffold levels 1 and 2 were determined and collected as scaffold libraries in NC-MFP. The selected scaffold libraries (676 scaffolds) generated from the DNP are summarized (Additional file 8).

The only way to increase both *DB coverage* and *accuracy in classification* in the NC-MFP was by supplementing the DNP contents. To increase structural discrimination between classes in DNP, (i) new representative compounds that positively contribute to the discrimination should be added to each class or (ii) the compounds that negatively contributed to the discrimination between classes should be removed. It was reasonable to add or remove representative compounds of each class in the DNP for increasing structural discrimination. By repeating the process of adding a representative compound that could play a role in removing the miss assigned compound represented by the off-diagonal element in the heat map to the original position of the diagonal element, one can achieve the optimum scaffold set for each class, and hence, can increase the *DB coverage* and *accuracy in classification*.

### Performance evaluation of molecular fingerprints by binary classification task I

In order to perform the binary classification task I, 1000 natural and 1000 synthetic compounds were selected from InterBioScreen (IBScreen) DB. To compare the ability of classification of NC structures in the NC-MFP with other molecular fingerprints, the binary classification task I of each fingerprint was trained with 1-Nearest Neighbors (1-NN) algorithm.

The average sensitivity and specificity of ten external validation data set were calculated to compare the performance among the molecular fingerprints. The performance results in task I of the molecular fingerprints are summarized in Table 4 (Additional file 9). The Y-randomization was used in the validation of models in the binary classification task I (Additional file 10).

**Table 4:** Binary classification result of task I^a^

Performance of each molecular fingerprint obtained by averaging ten external validation tasks^a,b^						
Molecular fingerprint	Natural compound classification			Synthetic compound classification		
	Avg. TP	Avg. FN	Avg. Sensitivity^c^ (%)	Avg. TN	Avg. FP	Avg. Specificity^d^ (%)
NC-MFP	183	14	92.65	113	87	56.50
MACCS	169	30	84.60	146	53	73.35
PubChem FP	165	34	82.60	154	46	77.00
GraphFP	161	38	80.75	143	56	71.80
APFP	153	46	76.55	141	58	70.70

^a^The result of performance about the binary classification task I. The external validation data set was randomly selected 10 times by a proportion of 20% from the data set. “NC-MFP” stands for Natural Compound Molecular Fingerprints and “APFP” for AtomPairs2DFingerprint and “GraphFP” for GraphOnlyFingerprint. “MACCS” reports Molecular Access System keys fingerprints and “PubChemFP” stands for PubChem fingerprint

^b^The performance index consist of Sensitivity and specificity. “TP” stands for True positive and “FN” stands for False negative and “TN” standards for True negative and “FP” standard for False negative

^c^The sensitivity is the proportion of positive class that was correctly identified

^d^The specificity is the proportion of negative class that was correctly identified

The sensitivity, the probability of accurately classify NC into NC, of the NC-MFP was obtained as 92.65%. The MACCS was obtained as 84.60%, which showed the second-best sensitivity. The lowest sensitivity was obtained with APFP at 76.55%. Unlike sensitivity, the specificity, the probability of accurately classify synthetic compounds into synthetic compounds, of the NC-MFP classification model was 56.50%, the lowest value among all fingerprints. In contrast, the models of the other fingerprints exhibited specificity larger than 70%.

NC-MFP consists of molecular scaffolds generated from the representative NCs of the classes of DNP compared to the aforementioned molecular fingerprints developed without distinguishing between synthetic and NC. Hence, the NC-MFP contains many distinct scaffolds suitable for expressing the characteristic structural fragments of natural products. Therefore, NC-MFP gives a better average sensitivity than the other molecular fingerprints. A large percentage of synthetic compounds share scaffolds with NC because many of the synthetic compounds have been synthesized via the modification of the molecular scaffolds or molecular structures of NC [49]. Since NC-MFP was developed based on the scaffolds of NCs, the specificity, the probability of classifying a synthetic compound as a synthetic compound, was observed to be lower than that of the molecular fingerprints developed with the molecular structure of synthetic compounds. Although NC-MFP shows low specificity, its ability to recognize NC as NC was observed to be better than the tested molecular fingerprints in this study.

In summary, NC-MFP has a disadvantage in terms of the ability to classify synthetic compounds as synthetic compounds from the data set. However, the high average sensitivity of NC-MFP suggests that the capability of the classification of NC structures from the data set is superior to the others. Since NC-MFP has the best average sensitivity in comparison with other molecular fingerprints, NC-MFP is a superior molecular fingerprint to classify structural differences or properties of NCs. Therefore, NC-MFP is a suitable molecular fingerprint for natural product research.

### Performance evaluation of molecular fingerprints using binary classification task II

Task I examined the ability of each fingerprint to distinguish between natural and synthetic compounds by analyzing the sensitivity and specificity of the classification model developed with each fingerprint. According to the analysis, NC-MFP showed the highest sensitivity and lowest specificity among the fingerprints introduced for the test.

The binary classification task II consisted of classifying whether the 897 NCs with biological activities against seven target proteins with inhibitory activity belonged to active or inactive class. Binary classification task II was carried out to evaluate the ability of molecular fingerprints to classify the NCs with biological activities as active or inactive. The performance of classification task II was measured with average accuracy (ACC) [47], F1-score [47, 50], and the Matthews Correlation Coefficient (MCC) [46] of ten external validation data sets for each target protein. Three evaluation indices have been generally used as standard methods of evaluation of binary classification [47]. The results of the performance of task II are reported in Table 5 (Additional file 11). To validate models of task II, Y-randomization was used (Additional file 12).

**Table 5:** Binary classification results of task II

Protein targets	Performance^a^ of each molecular fingerprint obtained by averaging ten external validation tasks^b^														
	NC-MFP			MACCS			PubChemFP			GraphFP			APFP		
	ACC^c^ (%)	F1^d^ (%)	MCC^e^	ACC^c^ (%)	F1^d^ (%)	MCC^e^	ACC^c^ (%)	F1^d^ (%)	MCC^e^	ACC^c^ (%)	F1^d^ (%)	MCC^e^	ACC^c^ (%)	F1^d^ (%)	MCC^e^
Protein-tyrosine phosphatase 1B (NPT 178)	78.98	80.65	0.57	66.90	72.56	0.32	69.66	74.40	0.36	67.24	71.88	0.33	61.03	58.07	0.29
Acetylcholinesterase (NPT 204)	73.42	76.42	0.49	70.79	75.75	0.42	70.00	76.15	0.41	66.58	72.05	0.30	59.74	63.94	0.18
Aldose reductase (NPT 68)	83.20	83.51	0.76	76.00	77.35	0.56	75.60	75.03	0.59	69.60	71.01	0.41	59.20	47.03	0.24
Beta-secretase (NPT 740)	87.20	88.64	0.83	77.20	80.48	0.55	73.20	77.46	0.45	77.20	81.44	0.53	71.20	74.78	0.48
Cyclooxygenase-2 (NPT 31)	84.76	86.37	0.78	74.28	79.30	0.56	69.52	74.69	0.45	73.33	77.36	0.45	63.81	60.26	0.35
Butyrylcholinesterase (NPT 439)	87.89	88.82	0.88	78.95	81.53	0.64	71.05	75.05	0.51	74.74	77.13	0.55	77.35	78.57	0.56
Cyclooxygenase-1 (NPT 324)	88.33	89.42	0.76	79.45	82.93	0.63	78.89	83.32	0.65	77.78	82.39	0.65	73.89	73.73	0.52
Average	83.40	84.83	0.72	74.80	78.56	0.53	72.56	76.59	0.49	72.35	76.18	0.46	66.60	65.20	0.37

The seven target proteins of task II and the compounds summarized in Table 1

^a^The performance index consist of accuracy (ACC), F1-score (F1) and the Matthews Correlation Coefficient (MCC)

^b^The result of performance about the binary classification task II. The external validation data set for each target is randomly selected 10 times from both active and inactive compound set of the target protein as of 20% in each target proteins. “NC-MFP” stands for Natural Compound Molecular Fingerprints and “APFP” for AtomPairs2DFingerprint and “GraphFP” for GraphOnlyFingerprint. “MACCS” reports Molecular Access System keys fingerprints and “PubChemFP” stands for PubChem fingerprint

^C^The accuracy (ACC) is the proportion of the total number of correct predictions

^d^F1-score (F1) is the harmonic average of precision and sensitivity

^e^Matthews Correlation Coefficient (MCC) is used to evaluate the binary classification performance. MCC has a range of − 1 to 1 where − 1 means a completely wrong binary classifier while 1 means an entirely correct binary classifier

The average accuracy of overall molecular fingerprints ranged from 66.60 to 83.40%. For NPT 324, NC-MFP showed the best average accuracy at 88.33% among the seven classification tasks. MACCS observed second-best average accuracy at 79.45%, while APFP showed the lowest average accuracy at 73.89%. The average accuracy of seven classification tasks with the NC-MFP were 78.98%, 73.42%, 83.20%, 87.20%, 84.76%, 87.89%, and 88.33% in NPT 178, 204, 68, 740, 31, 439, and 324 respectively. NC-MFP showed high average accuracy in seven classification tasks and other molecular fingerprints.

The average F1-score was observed between 65.20 and 84.83%. For NPT 324, the average F1-score with NC-MFP was 89.42% as compared to other molecular fingerprints. MACCS showed a second-best average F1-score at 82.93%, and the difference between NC-MFP and MACCS was 6.49%. Each of the average F1-score from the seven classification tasks with the NC-MFP showed 80.65%, 76.42%, 83.51%, 88.64%, 86.37%, 88.82%, and 89.42% for NPT 178, 204, 68, 740, 31, 439, and 324, respectively. The overall F1-score of NC-MFP was observed to be more than 76%, and it outperformed all the other molecular fingerprints in each of the seven classification tasks.

The MCC values of overall molecular fingerprints ranged from 0.37 to 0.72 for seven classification tasks. Each of the average MCC for the seven classification tasks with NC-MFP showed values of 0.57, 0.49, 0.76, 0.83, 0.78, 0.88, and 0.76 for NPT 178, 204, 68, 740, 31, 439, and 324, respectively. For NPT 439, the average MCC with NC-MFP showed the best average at 0.88 compared with the other molecular fingerprints. Except for NPT 178 and 204, more than 0.75 overall average MCC of seven classification tasks with NC-MFP showed an overall excellent performance. Although the MCC value of NC-MFP was lower than 0.7 in NPT 178 and 204, NC-MFP outperformed the other molecular fingerprints. In comparison with the other molecular fingerprints, NC-MFP showed high average MCC for each of the seven classification tasks than the other molecular fingerprints.

NC-MFP showed the best performance in comparison with other molecular fingerprints in the overall performance of task II. The best performance of NC-MFP is construed as meaning that the classification of NCs with inhibitory activities on seven target proteins is entirely accurate. Moreover, it also suggests that the structural features of the NC-MFP correlate with biological activities and explain them well.

### Comparison between NC-MFP and other molecular fingerprints

Based on the result of two binary classification tasks, the overall performance of MACCS and PubChemFP was noted to be relatively lower than NC-MFP. Since MACCS and PubChemFP focused on structures of synthetic compounds; therefore, it is difficult to classify the structural differences among NCs. Besides, structural features of MACCS and PubChemFP show a small size. Since the structural features of small size can be included in complex NC structures, it is challenging to represent the precise NC structure. APFP and GraphFP mainly focus on chemical connectivity information of synthetic compounds. Since the NC structures have complicated fused ring system and complex fragments compared to the synthetic compounds, it may not be a good approach to apply to NC structures.

However, since the NC-MFP was composed of structural features derived from NCs and structural features, it could correlate with biological activities, and NC-MFP showed the best performance when applied to NC structures compared with other molecular fingerprints relatively.

In summary, this study provides the novel molecular fingerprint optimized to NC structures. We show that the NC-MFP is a more competent molecular fingerprint to describe NC structure and to explain the correlation between NC structures and biological activities on target proteins when compared with other molecular fingerprints. Furthermore, since high accuracy is of significant interest in the industrial sector, NC-MFP can be a powerful tool to screen NC structures for determining new candidate drug structures with high accuracy. Moreover, it can be used as a valid tool as a molecular descriptor for NP-based new drug development. Also, it is expected to be an appropriate molecular descriptor for virtual screening of NP-based new drug development.

## Conclusion

In this study, we introduced NC-MFP based on the structural characteristics of NCs. NC-MFP is a scaffold-based molecular fingerprint that utilizes the DNP’s classification system of 16 classes. The scaffold of NC-MFP was generated from representative compounds of each class in DNP using the BM method. Since NC-MFP depends on the contents of the DNP, it is difficult to cover the molecular structures of all the known NCs. By repeating the process of adding or removing representative compounds that can contribute to the discrimination of each class in DNP, the DB coverage of NC-MFP could reach close to 100%.

Two types of binary classifications tasks were performed with 1-NN to evaluate the performance of NC-MFP compared to other molecular fingerprints. NC-MFP showed the best performance as a result of two binary classification tasks. We show that NC-MFP is a robust molecular fingerprint in classifying NC structures and explaining biological activities on target proteins. Therefore, we conclude that the NC-MFP is specially designed for NC structures and is a new molecular fingerprint for virtual screening of NC structures. Furthermore, since the NC-MFP is a descriptor for virtual screening of NC structures with biological activities, it would be applied as a competent method for developing new drugs based on NC structures.

## Supplementary information

**Additional file 1.** Chemical compounds of the Natural Compound Databases (NCDBs).

**Additional file 2.** Python code to generate NC-MFP algorithm and select optimized scaffolds.

**Additional file 3.** Chemical compounds of the binary classification task I.

**Additional file 4.** Binary classification task I model of RapidMiner Studio 9.2 and molecular fingerprint data set to test model.

**Additional file 5.** Chemical compounds of the binary classification task II.

**Additional file 6.** Binary classification task II model of RapidMiner Studio 9.2 and molecular fingerprint data set to test model.

**Additional file 7.** 16 classes of representative compounds in DNP.

**Additional file 8.** Optimized scaffold libraries produced with DNP by using the BM method in pipeline pilot 2017.

**Additional file 9.** External validation results of the binary classification task I.

**Additional file 10.** Y-randomization results of the binary classification task I.

**Additional file 11.** External validation results of the binary classification task II.

**Additional file 12.** Y-randomization results of the binary classification task II.

## Data Availability

All data generated or analyzed during this study are included as the additional information to the article. The python code of the NC-MFP algorithm using the RDKit python package is provided in additional file. The binary classification task models and data set are provided in additional file. Requirements: Window OS, an RapidMiner Studio 9.2.

## Abbreviations

NC-MFP: Natural Compound Molecular Fingerprint
DNP: Dictionary of Natural Products
NC: natural compounds
NP: natural product
SFCP: scaffold-fragment connection point
BM: Bemis and Murko
1-NN: 1-Nearest Neighbor
MACCS: Molecular ACCess Systems keys fingerprint
PubChemFP: PubChem Fingerprints
APFP: AtomPairs2DFingerprint
GraphFP: GraphOnlyFingerprint
MCS: Maximum Common Substructure
MMP: Matched Molecular Pairs
NCDBs: Natural Compound Databases
IBScreen: InterBioScreen
NPACT: Naturally occurring Plant based Anticancerous Compound-Active-Target Database
TCM: Traditional Chinese Medicine
NPASS: Natural Product Activity and Species Source Database
k-NN: k-Nearest Neighbors algorithm
MCC: Matthews Correlation Coefficient
